# Transforming stress program on medical students’ stress mindset and coping strategies: a quasi-experimental study

**DOI:** 10.1186/s12909-023-04559-9

**Published:** 2023-08-18

**Authors:** Tan Nguyen, Christy Pu, Alexander Waits, Tuan D. Tran, Tuan Hung Ngo, Quynh Thi Vu Huynh, Song-Lih Huang

**Affiliations:** 1 Institute of Public Health, National Yang Ming Chiao Tung University, Taipei, Taiwan; 2 University of Medicine and Pharmacy, Ho Chi Minh City, Vietnam; 3 Medigen Vaccine Biologics, Taipei, Taiwan

**Keywords:** Stress mindset, Coping strategies, Medical students, Stress intervention, Experimental study

## Abstract

**Objective:**

Stress is a significant concern in medical education, and identifying effective ways to deal with stress may help with students’ mental health and professional development. This study aimed to examine the effects of the Transforming Stress Program (TSP) amongst first-year medical students on their stress mindset and coping strategies when confronted with stressors.

**Methods:**

We conducted a quasi-experimental study at the University of Medicine and Pharmacy, Ho Chi Minh City, Vietnam. A total of 409 first-year students at the Faculty of Medicine were divided into intervention group (205 students) and control group (204 students). The 10-week TSP was delivered as an extra-curricular course. The training adopts psychoeducation based on Dialectical Behavioral Therapy with mindfulness as a fundamental practice incorporated into each component of the program. The intervention group received the training in the first semester; the control group received identical program in the second semester. Stress Mindset Measurement and Brief Coping Orientation to Problems Experienced were measured before the intervention (T0), immediately after intervention on Intervention group (T1), and six months after intervention on Intervention group (T2).

**Results:**

At T1, the intervention group showed 65% improvements in stress mindset scores and increases in coping strategies scores in six domains (Problem solving, Social support, Humor, Religion, Venting, and Self-distraction) and decreases in three (Avoidance, Substance use, and Self-blame). The effect sizes were significant in all outcomes (Cohen’s d > 0.2). Measurements of the control group did not change significantly in the same period. At T2, effects of the TSP were found decreased in some domains (Avoidance, Substance use, and Self-blame) compared to T1, but largely remained significantly better than T0.

**Conclusions:**

The TSP is a feasible and effective approach that significantly enhanced medical students’ stress mindset and coping strategies. Some effects were still observable 6 months after the intervention. The relatively intensive intervention requires support of the school administration and staff.

**Supplementary Information:**

The online version contains supplementary material available at 10.1186/s12909-023-04559-9.

## Introduction

Stress is a significant concern in medical training. Stressors come from academic workload, high self-expectation, financial burdens, and difficulties in working with seniors and patients in the clinical settings [1–3]. Prolonged stress influences students’ performance in schools, and later can lead to professional misconducts [4, 5]. There are different factors contributing to professional misconduct among students with distress. Stress may negatively affect students’ academic performance, which may lead to future poor clinical performance in the workplace setting. Previous studies also reported that prolonged stress may cause a decline in professionalism in patient care. Therefore, being exposed to excessive stress may lead to professional misconduct among medical students [5]. Moreover, studies show that high levels of stress in medical students lead to decreased empathy level in doctor-patient relationships and affect the quality of healthcare. Other negative consequences include mental health issues, substance use, and suicidal ideation and attempts [6]. Previous surveys among medical students in Vietnam reported prevalence of stress to be around 50%, while prevalence of depression ranged from 25% to above 50%. Furthermore, suicidal ideation was reported among 5.8–7.7% [7–9].

Over the past twenty years, stress management programs in medical training have gained increasing attention from medical educators and administrators [10–13]. Earlier interventions focused on methods to minimize stressful experiences, namely, intensity, frequency, and the duration of stressors, as well as techniques of relaxation and meditation when facing stressors [14–16]. Recent research shifted emphasis to the role of stress mindset in moderating the impact of stressors on health and productivity. In one study of Crum et al. in 2013, participants were randomized into “stress-is-enhancing” group, “stress-is-debilitating” group, and control group. Participants in the “stress-is-enhancing” group and “stress-is-debilitating” group were given three different videos about the effect of stress on health, on work-performance, and on learning/growth. Participants in the control group did not view any video. These videos were composed of words, music, and images [17]. After the intervention, participants in the “stress-is-enhancing” group showed increased stress mindset scores, while participants in the “stress-is-debilitating” group showed decreased stress mindset score. There was no significant change of stress mindset score in the control group. Findings also revealed that participants of the “stress-is-enhancing” groups decreased their scores of psychological symptoms, while increased work performance in both soft skills and hard skills. During the same period, there were no significant changes in these aspects in the other two groups. In 2023, Crum conducted another study on the effects of a 2-hour training named “Mindset Training Program”, which provided participants with knowledge about the nature of stress, both enhancing and debilitating. The participants were given a workbook to practice self-reflection afterwards, and were taught different strategies to adopt the stress-is-enhancing mindset in order to utilize the good side of stress [18]. After the intervention, in the intervention group, stress mindset score increased significantly with marginal increase in their work performance. In 2019. Keech conducted a study to evaluate the effect of changing stress mindset by an imagery intervention. Participants watched a series of videos, including balanced information about stress and the consequences of stress (both negatively and positively), and mental imagery exercise on the positive consequence of stress [19]. Findings from Keech’s study also demonstrated the effect of a mindset intervention on enhancing stress mindset. This effect was still observable after following up for two weeks.

Research showed that the way we perceive, evaluate, and cope with stressors would greatly influence downstream outcomes [17, 20−22]. Stress mindset is conceptualized as the extent to which an individual holds the mindset that stress has either enhancing or debilitating consequences for various stress-related outcomes, resulting in different ways of appraising stress as either a challenge or a threat [17, 23]. Evidence shows that stress mindset is related to outcomes of stress, including health and well-being, learning and growth, as well as performance and productivity [20, 22, 24]. Intervention on stress mindset may be a promising approach to improve people’s response in the face of stress, which in turn would improve the ultimate outcomes in health, and work-related performance [17, 25].

Coping strategies are also important in shaping the association between the experience of stress and mental well-being [26, 27]. When confronted with stressors, coping strategies are people’s cognitive and behavioral efforts to overcome difficult situations. Studies suggest that students’ coping strategies during preclinical years would affect their future career satisfaction and ability to resist burnout [28, 29], implicating the essential role of coping skills training for medical students. Studies emphasized the importance of enhancing coping strategies for medical trainees, particularly during their early years of training, as adaptive coping strategies are associated with lower levels of burnout, emotional distress, and higher sense of accomplishments [30, 31].

Prior studies of stress management programs had inconsistent results, probably because of methodological weaknesses including the lack of control group and follow-up [32, 33]. Therefore, studies with well-validated assessment tools and appropriate design are in need to further validate the effectiveness of stress interventions, particularly in middle-income countries, where evidence is insufficient. In Vietnam, only one study reported an increase in medical students’ quality of life after receiving a mental well-being program. This program included activities in three levels as listed: intrapersonal, interpersonal, and institutional level. On an intrapersonal level, they provided students with skill training, focusing on time management skills, communication skills, problem-solving skills, and stress management skills. On an interpersonal level, activities were held to enhance students’ sense of connection and belonging, which were activities to connect students with seniors and Student Association Unit. On an institutional level, they promoted counselling services and a mobile phone application to make counselling more accessible to students. Results showed students in intervention group had better scores in their well-being when compared to students in control group [34].

To fill the above gap, this study aims to examine the effects of the Transforming Stress Program (TSP) amongst first-year medical students in University of Medicine and Pharmacy, Ho Chi Minh City (UMP). The TSP is a self-developed training program, adopting psychoeducation to reframe the stress mindset, and provide students with adaptive coping strategies. Findings from this study will contribute to the empirical evidence of effectiveness of the stress management programs in Vietnam and other low- and middle-income countries.

## Methods

### The intervention: the transforming stress program (TSP)

The program was developed by the research team based on existing stress management programs for students [11, 35−37]. Our team included the principal investigator and two psychologists. After doing literature review on stress management programs, we decided to adopt approaches that work with students’ mindset and their coping strategies. Cognitive Behavioral Therapy (CBT) and Dialectical Behavioral Therapy (DBT) were chosen because they have been utilized in adjusting people’s ways of thinking and behaving. We then built the three core objectives of the TSP, which were transforming stress mindset, improving adaptive coping strategies, and maintaining their practice after the training. The two psychologists worked on the content of each training session to achieve the training objectives. The principal investigator worked with the school leaders and school administrators to arrange the schedule for the TSP to be incorporated into the first-year student curriculum. We also had pilot training on 37 first-year students and their feedback was incorporated into the final TSP.

CBT and DBT models were applied to help participants recognize their stress mindset, immediate thoughts, emotions, and behaviors. DBT was shown to be effective in addressing different mental health and behavioral problems, particularly its function of enhancing people’s capabilities by promoting emotional regulation skills, interpersonal skills, and distress tolerance skills. While CBT focused on transforming students’ stress mindset into stress-is-enhancing mindset, we applied DBT to improve their coping strategies when confronting stressors [38]. Students then could label stress triggers, regulate their emotions, and make personal plans to cope with stress. Post-stress self-reflection, deep breathing, paired muscle relaxation, asking for help in time of need, writing gratitude journal, and developing new habit to take care of body and mind are the core coping skills that students were provided and encouraged to keep practicing. Mindfulness was fundamental practice and was incorporated into each component [39–43].

Through formal lectures, group discussion, problem-based learning, experience sharing, and following up by instructors, participants are encouraged to transform their stress mindset into “stress is enhancing” rather than “stress is debilitating”. Students were also provided with a variety of adaptive coping strategies. The TSP was held in five sessions; the first three were theory sessions (two hours each), and the last two were follow-up sessions (one hour each). Students were encouraged to practice between sessions and share their experience during the follow-up sessions facilitated by instructors. Instructors supported students throughout the intervention period via social media with recommendations for their challenges and concerns. The two instructors coordinated their activities and comments so to minimize variations. (The detailed course content of TSP is attached in Additional file 1_Transforming Stress Program’s Components)

### Study design

**Fig. 1:**
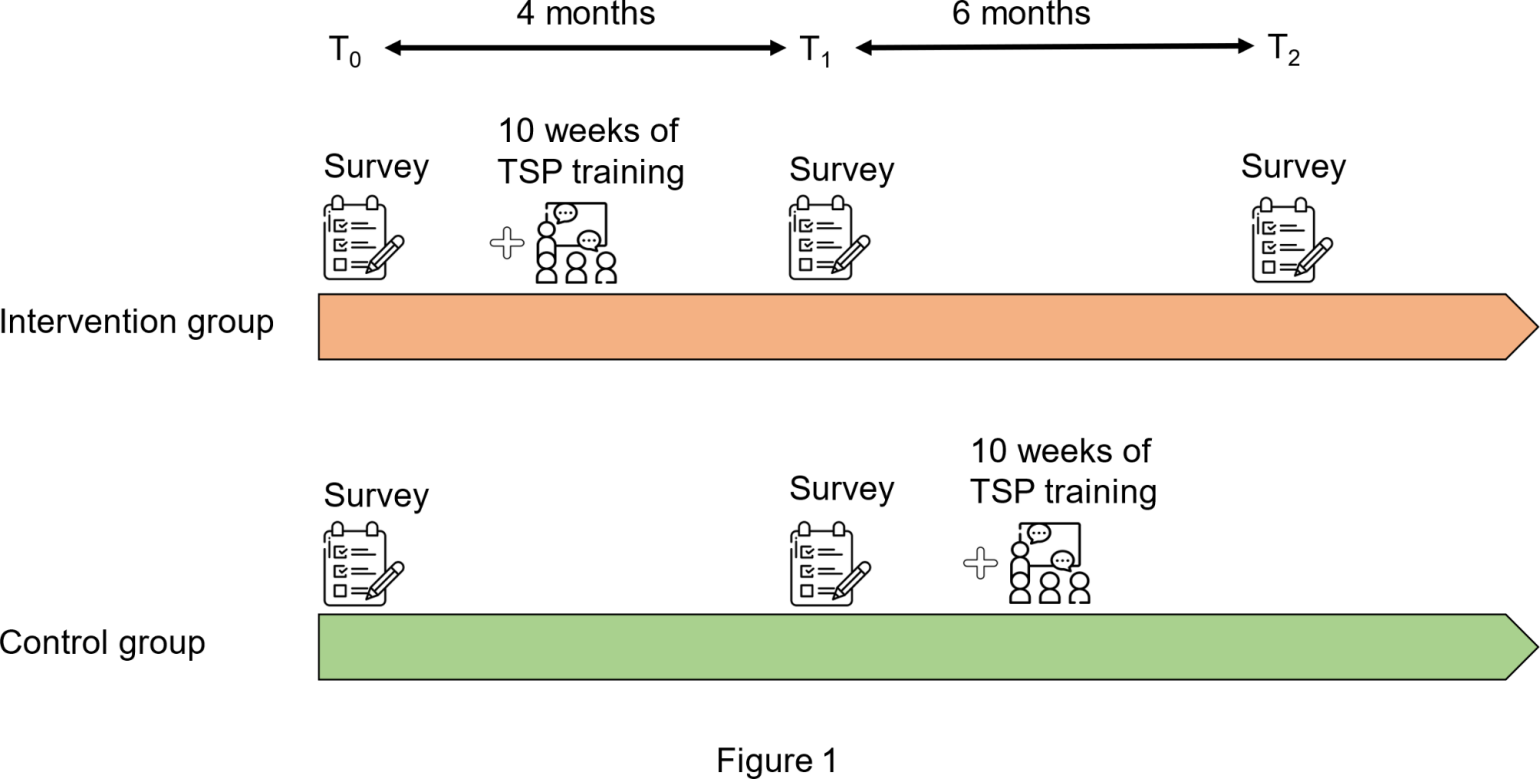
Timeline of TSP and measurements in the intervention group and control group. TSP: transforming stress program. T0: before intervention on the Intervention group, T1: immediately after intervention on the Intervention group, T2: six months after intervention on the Intervention group Survey at T0: demographic characteristics, SMM, brief COPE. Survey at T1, T2: SMM, brief COPE

A quasi-experimental study was conducted. A pilot study with 37 students was carried out to assess the feasibility of the TSP and for instrument validation. The intervention was modified based on feedbacks. The 10-week TSP was delivered to the intervention group in the first semester. In the second semester, the control group received the identical program. During the first semester, the control group served as the wait list control. We collected data at T1, immediately after the intervention on the intervention group, but not yet on the control group. The differences of the outcomes from T0 to T1 between the intervention group and control group would be analyzed to show the effects of the TSP. In Fig. 1, T1 was around the end of the first semester. Hence, the intervention group received the TSP training during the first semester (before T1), while the control group received the intervention in the second semester (after T1). Contamination may happen if students in the intervention group shared with students in control group about the TSP training, hence, we divided the intervention group and control group along the line of their class assignment. Because interaction among students in the same class was much more intensive than with those across classes, this design may minimize contamination between the two groups. However, any contamination will reduce, rather than increase, the estimated effect of the intervention. The intervention group was followed-up for a six-month period after the completion of the intervention (see Fig. 1).

### Participants

Out of 420 first-year students at the Faculty of Medicine, 409 students agreed to participate in the study (participation rate 97%). Meanwhile, 11 students did not participate due to time conflicts with their personal schedules. The participants were divided into intervention and control groups based on their class assigned by the school, with 205 students in the intervention group and 204 students in the control group. The class assignment is based on students’ entrance exam scores and sex, with even distribution in mind.

### Procedures

The principle investigator introduced the study in an orientation session attended by all first-year students. It is mandatory for first-year students to take extra-curricular courses; TSP was listed as one such course for this academic year and students were encouraged to take it and earn credits for the extra-curricular activities. The participants submitted written informed consent before participating in the TSP course.

The baseline data for both groups was collected before the intervention (T0), and the second data collection for both groups was conducted after the intervention group completed the training (T1). The follow-up measurements (T2) were conducted six months after T1 (see Fig. 1). The data was collected through online self-report questionnaires with paper-pencil forms on request. Surveys at T0 consisted students’ demographic data, stress status, Stress Mindset Measurement (SMM), and Brief Coping Orientation to Problems Experienced (Brief COPE).

### Instruments and measurements

Stress mindset was measured by SMM, an eight-item self-report scale [17]. The score is between 0 and 4; a higher score corresponds to the “stress-is-enhancing” mindset. SMM in this study population had an acceptable internal consistency (Cronbach’s alpha = 0.75).

Coping strategies were measured by Brief COPE [44, 45] to assess individuals’ ways of coping with stressors. Factor analysis of data from this study suggested a revised inventory with 27 items, excluding item 20. The 9-factor structure had acceptable to good fits, including Problem-solving (7 items), Social support (4 items), Avoidance (4 items), Substance Use (2 items), Self-blame (2 items), Humor (2 items), Religion (2 items), Venting (2 items), and Self-distraction (2 items). Responses to the items were scored using a four-point Likert-style scale ranging from 1 (I usually don’t do this at all) to 4 (I usually do this a lot). A higher score represents a higher frequency of using that strategy.

This study was approved by the IRB of both National Yang Ming Chiao Tung University and UMP, and an applicable guidelines for research with human subjects were followed.

### Statistical analyses

We used RStudio [46] for data analyses. Descriptive statistics were presented by mean and standard deviation for continuous variables, and frequency and percentage for categorical variables.

The four-month effects of the intervention were examined by analyzing the difference in outcomes from T0 to T1 between the intervention group and control group. We used linear regression models with intervention as a dummy variable. Univariate and multiple linear regressions were conducted to investigate the effects of intervention controlling for students’ demographic factors (sex, age, parental education, part-time job), stress-related factors (Covid-related stress, physical or psychological issues, acute stress event), or their levels of engagement during the intervention. The ten-month effects were measured only in the intervention group, after six months of follow-up. Paired T test was used to compare the scores of stress mindset and nine coping strategies between T2 and T1 in the intervention group. Paired T test was also utilized to compare these outcomes between T0 and T1 in both intervention and control group.

## Results

### Baseline characteristics of the participants

Table 1 presents baseline demographic characteristics of the 409 participants. Approximate 38% of the participants were female. More than 50% of their parents finished college or higher. The study was initiated in November 2020, before there was a surge in cases in 2021 which urged the government to issue a strict quarantine policy. Hence, only about 10% of students reported being stressed due to Covid-19. The intervention and control groups had similar demographic characteristics, except for mothers’ education status (p value < 0.01). At baseline, the most frequently used coping strategies were Self-distraction, Problem solving, and Social support, while Substance use was the least common strategy adopted by the participants. The pattern was similar for the two groups. However, the control group had higher Problem solving and Self-distraction scores and lower Avoidance score.

**Table 1:** Baseline characteristics of the participants

	Intervention group (N = 205)	Control group (N = 204)	P value
Age			0.360
Mean (SD)	18.1 (0.34)	18.1 (0.42)	
Sex, N (%)			0.710
Female	78 (38.0%)	73 (35.8%)	
Father education, N (%)			0.237
Elementary or lower High school College or higher	3 (1.5%) 58 (28.3%) 144 (70.2%)	8 (3.9%) 50 (24.5%) 146 (71.6%)	
Mother education, N (%)			0.007
Elementary or lower High school College or higher	7 (3.4%) 69 (33.7%) 129 (62.9%)	14 (6.9%) 92 (45.1%) 98 (48%)	
Part-time job, N (%)			0.202
Yes	20 (9.8%)	12 (5.9%)	
Physical stress, N (%)			0.707
Yes	47 (22.9%)	51 (25.0%)	
Psychological stress, N(%)			1,000
Yes	47 (22.9%)	46 (22.5%)	
Covid stress, N (%)			0.987
Yes	19 (9.3%)	20 (9.8%)	
Stress level, N (%)			0.759
Low Moderate High	71 (34.6%) 118 (57.6%) 16 (7.8%)	71 (34.8%) 113 (55.4%) 20 (9.8%)	
Stress mindset			0.078
Mean (SD)	1.78 (0.52)	1.87 (0.60)	
Problem Solving			0.012
Mean (SD)	2.65 (0.55)	2.78 (0.5)	
Social Support			0.307
Mean (SD)	2.58 (0.66)	2.65 (0.73)	
Avoidance			0.024
Mean (SD)	1.94 (0.53)	1.81 (0.57)	
Substance Use			0.230
Mean (SD)	1.30 (0.56)	1.23 (0.52)	
Self-blame			0.592
Mean (SD)	2.54 (0.70)	2.50 (0.77)	
Humor			0.731
Mean (SD)	2.07 (0.64)	2.04 (0.64)	
Religion			0.123
Mean (SD)	1.81 (0.82)	1.94 (0.81)	
Venting			0.079
Mean (SD)	2.44 (0.72)	2.56 (0.69)	
Self-distraction			0.016
Mean (SD)	2.72 (0.68)	2.88 (0.67)	

### The four-month effects of the transforming stress program

Table 2 shows the scores of stress mindset and the nine coping strategies among students in both intervention and control group before and after the intervention group received the TSP. In the intervention group, the scores of stress mindset and eight coping strategies (except for Substance use) were statistically different between before and after receiving the TSP training. The mean score of stress mindset increased by approximately 65%. The scores also increased in six domains of adaptive coping strategies: Problem solving (by 20%), Social support (by 22%), Humor (by 20%), Religion (by 17%), Venting (by 20%), and Self-distraction (by 8%). In contrast, the mean scores of maladaptive coping strategies dropped, ranging between 5% in Substance use to 25% in Self-blame. On the other hand, during the same period, among students in the control group, only the score of Substance use was found to significantly increase. The difference between the two groups suggests that the change we observed in the intervention group was the result of TSP, rather than some learning process or life-style changes brought about by the first year of medical education. The significance of these differences between the two groups is examined below (Table 3).

**Table 2:** The scores of stress mindset and coping strategies before and after the TSP^a^

	Intervention			Control		
	T0	T1	P value	T0	T1	P value
Stress mindset	1.78	2.93	< 0.001	1.87	1.87	0.817
Problem solving	2.65	3.18	< 0.001	2.78	2.73	0.136
Social support	2.58	3.15	< 0.001	2.65	2.62	0.538
Avoidance	1.94	1.60	< 0.001	1.81	1.88	0.190
Substance use	1.30	1.23	0.173	1.23	1.4	< 0.001
Self-blame	2.54	1.90	< 0.001	2.50	2.4	0.152
Humor	2.07	2.49	< 0.001	2.04	2.15	0.105
Religion	1.81	2.11	< 0.001	1.94	1.95	0.877
Venting	2.44	2.94	< 0.001	2.56	2.48	0.225
Self-distraction	2.72	2.95	< 0.001	2.88	2.86	0.733

^*a*^ *The range of stress mindset: 0–4, the range of coping strategies: 1–4*

*T0: before TSP was delivered on the intervention group, T1: after TSP* was delivered on the intervention group

Table 3 shows the effects of TSP on stress mindset and coping strategies at the completion of the intervention; linear regression coefficients and Cohen’s d were used to estimate the effects and effect sizes. The analyses were adjusted for students’ demographic characteristics, stress-related factors (acute event stressor, Covid-related stress, psychosocial stress, physical stress), and the level of participant’s engagement in the TSP. For the stress mindset, the coefficient in the linear regression showed a significant increase of the score by 1.16 point (within the range between 0 and 4). In terms of adaptive coping strategies, within the range between 1 and 4, the coefficients indicated increased scores of Problem solving (by 0.59 point), Social support (by 0.6 point), and Venting (by 0.58 point). With a lesser extent, the scores of Humor, Religion, and Self-distraction increased by 0.48 point, 0.29 point, and 0.25 point, respectively. For the three maladaptive coping strategies, including Avoidance, Substance Use, and Self-blame, their scores significantly changed in the negative direction. The Cohen’s d indices are significant in all outcomes (d > 0.2), with the largest size effects found on Stress mindset, and Problem solving.

**Table 3:** The difference of changes in outcomes between the Intervention group and Control group at four-month

	Coef (SE)^a,b^	Cohen’s d
Stress mindset	1.16 (0.08) ***	1.38
Problem solving	0.59 (0.07) ***	0.86
Social support	0.60 (0.09) ***	0.67
Avoidance	-0.40 (0.08) ***	-0.56
Substance use	-0.23 (0.08) ***	-0.33
Self-blame	-0.54 (0.10) ***	-0.56
Humor	0.48 (0.11) ***	0.34
Religion	0.29 (0.11) **	0.29
Venting	0.58 (0.09) ***	0.62
Self-distraction	0.25 (0.09) **	0.28

^*a*^ *The range of stress mindset: 0–4, the range of coping strategies: 1–4*

^*b*^ *Model adjusted for demographic factors, Baseline stress level, Acute stress event, Physical issue, Psychological issue, Covid-19 related stress, and Class engagement level*

Signif. Codes: *p < .05. **p < .01. ***p < .001

### The ten-month effects of the TSP

Table 4 shows the changes in outcomes six-month after the completion of TSP in the intervention group. Paired t test indicated significant changes of stress mindset and seven of the nine domains of coping strategies, except for Religion and Self-distraction. The mean difference of stress mindset score was 0.16, a 6% decrease from T1 to T2. For adaptive strategies, the scores of Problem solving, Social support, and Venting decreased by 0.72 point (23%), 0.46 point (15%), and 0.14 point (5%), while the score of Humor increased by 0.52 point (21%). Regarding maladaptive strategies, the scores of Avoidance, Substance use, and Self-blame were higher at T2, particularly the score of Substance use increased by 87% (1.07 point). In short, amongst the participants in the intervention group, the training effects reduced in magnitude for Stress mindset, and coping strategies, including Religion and Self-distraction. The effects diminished by time in the domains of Problem solving, Social support, Venting, Avoidance, Self-blame, and particularly Substance use. Yet, the effect increased significantly in one domain – Humor. Nevertheless, when compared to baseline, the scores at T2 remained significantly higher for stress mindset and most coping strategies, except for Social support and Avoidance domains.

**Table 4:** The ten-month effects of TSP on the outcomes in the intervention group

Paired t test	Mean of differences, T2 – T1 (95%CI)^c^	Mean of differences, T2 – T0 (95%CI)^d^
Stress mindset	-0.16 (-0.21 – -0.11)***	0.99 (0.91–1.07)***
Problem solving	-0.72 (-0.82 – -0.62)***	-0.19 (-0.28 – -0.10)***
Social support	-0.46 (-0.57 – -0.35)***	0.11 (-0.04–0.26)
Avoidance	0.34 (0.25–0.42)***	0.002 (-0.10–0.10)
Substance use	1.07 (0.96–1.17)***	1,00 (0.90–1.10)***
Self-blame	0.17 (0.08–0.26)***	-0.47 (-0.59 – -0.34)***
Humor	0.52 (0.40–0.64)***	0.95 (0.82–1.08)***
Religion	-0.002 (-0.09–0.09)	0.30 (0.17–0.42)***
Venting	-0.14 (-0.24 – -0.04)**	0.37 (0.24–0.49)***
Self-distraction	-0.05 (-0.16–0.05)	0.18 (0.06–0.29)**

^*c*^ *Mean of differences: the differences in the scores measured at ten-month and at four- month after receiving TSP, examined by paired t test*

^*d*^ *Mean of differences: the differences in the scores measured at ten-month after receiving TSP and baseline, examined by paired t test*

Signif. Codes: *p < .05. **p < .01. ***p < .001

## Discussions

This study examined the effects of a ten-week program on medical students’ stress mindset and coping strategies in an Asian country; it included a control group and a follow-up period of 6 months. Results showed improvements in stress mindset and all coping strategies immediately after the completion of TSP, and most of these effects were still observable six months later. Further, the effects were not associated with students’ demographic characteristics, their stressors, or engagement in the TSP. The control group received the TSP in the second semester as it was provided as an extra-curricular program to all students, but they were not exposed to TSP when measurements were taken at T1.

### The effects of the TSP on stress mindset

Immediately after the completion of TSP, there was a substantive improvement of stress mindset score (by 65%), demonstrating a remarkable shift from “stress-is-debilitating” to “stress-is-enhancing” mindset. This effect diminished over time, with the score decreased by 6% after six months, yet the score was still higher than the baseline score (2.77 at T2, 2.93 at T1, and 1.78 at T0). These findings provide further evidence that mindset can be altered by orienting participants to different understanding of stress [17]. In the current study, the intervention adopted a psychoeducation approach to bring about changes. This approach added a few elements to existing method of in-class lecture and discussion: interactive cellphone texting with instructors, short videos, and cellphone-based stress test to provide feedback to the participants [22, 37, 47]. Despite of these modifications, the interventions followed the same underlying theory as previous studies to provide the participants with the true nature of stress, emphasizing the enhancing nature of stress, and positive effects of having a “stress-is-enhancing” mindset [17, 22]. Studies of stress mindset intervention have largely been conducted in the United States and western countries, with the longest follow-up period of three months after the intervention [17, 23, 48]. Our results showed that alterations in stress mindset could be maintained six months after the intervention, and that the program was effective among medical students.

### The effects of the TSP on coping strategies

We found that TSP increased the use of the adaptive coping strategies immediately after the intervention, with simultaneous reduction in the use of maladaptive coping strategies. Most of these effects were substantial with 20–25% changes from baseline. These results were encouraging since training programs of coping strategies for medical students have not been consistently proven effective [12, 49, 50]. For example, a study of 38 Spanish psychology students showed that mindfulness training helped improve problem-based and emotional-based copings, but the effects were only found in students with medium to high level of self-regulation [50]. Mentoring program was also found effective in improving nursing students’ self-confident and seeking social support [51]. A course aimed at improving students’ self-awareness and communication techniques helped them cope better by increase their sense of humor and self-confidence [52]. This study complemented the positive findings of previous ones, as each was based on different theoretical background, intervention approach, and outcomes measurements [50–52]. The TSP based on Dialectical Behavioral Therapy, which incorporated mindfulness practice, and stress mindset intervention into training for coping strategies; we demonstrated that this combination of approaches was feasible and effective. We used Brief COPE to measure outcomes because its categories of coping correspond with the TSP training content and best suited for the evaluation.

In Table 2, the significant increase in frequency of Substance use was reported only in the control group, but not in the intervention group. Though students in the intervention group received the TSP training, their scores in Substance use after receiving the training did not significantly decrease. It is likely that during the first year of college life, many students will pick up the habit of smoking and/or drinking. This may explain why we observe a significant increase of Substance use score in the control group. The results showed that the intervention could slow this increase, at least temporarily, perhaps by reducing the detrimental effects of stress, or by directing students to cope with stress by other means. However, after six months following up of the intervention group, the effects reduced in stress mindset and most domains of coping strategies with a marked increase in the score of substance use by 87%. In the field of intervention studies on coping strategies among medical students, we found one study with six years of follow-up period. In addition to a seven-week mindfulness-based course, booster sessions twice a year were provided; participants were able to maintain their practice on problem-focused coping [12]. In the current study, we observed significant increase in the adoption of dysfunctional coping strategies during the follow-up period, probably indicating the importance of continual reinforcement after the TSP. This observation could be also attributed to the surging wave of Covid-19 pandemic around T2, when participants faced lots of unexpected stressors. The wave of Covid-19 pandemic also influenced our data collection process, so while most participants finished the follow-up survey within the designated month, a minority (28 out of 205 students in the intervention group) stretched to the third month.

The relative ineffectiveness of short-term interventions on substance use was consistent with previous studies, demonstrating either small effect size or no effect on the use of alcohol, tobacco, and other illicit drugs [53–56]. Moreover, the TSP did not focus specifically on substance use or on students with high risk of substance use [57]. It did not address both risk factors and protective factors [58, 59], and it could not take into account family-based factors influencing students’ substance use. Also, most school-based program on substance use required an average duration of three years to be effective [60]. As substance use is a critical issue in medical students and physicians, further effective intervention programs may need to consider the above factors.

The contribution of the current study is that it provides another piece of evidence on the effects of a particular intervention on improving medical students’ stress mindset and coping strategies, independent of many background factors and baseline stress levels. Studies suggested that the change of stress mindset could be internalized and subsequently transform people’s behaviors, including coping strategies [17, 22]. However, because TSP included both intervention on stress mindset and coping strategies, it is impossible to delineate the effect of these components on the improvement of coping skills.

The TSP was offered as an extra-curricular course for all first-year students at the Faculty of Medicine. This goes well with the trend of medical education worldwide to develop curricular changes to address medical student stress and psychological health. Our study has the sample size of 409, much higher than other studies on coping training with sample size ranging from 10 to 57 [15, 16, 43, 50, 61]. The practicability of the intervention was in part dependent on the support from the President of the University, the Dean of the Faculty, and the school staff, demonstrating the importance of commitment from school administration.

Further studies may need to investigate the effects of the TSP on medical students of different years of training. It may also be necessary to examine if the content of the training needs to be adjusted to better fit the needs of different stages in medical training, such as third-year or fifth-year in medical school. The other area that requires continual attention is to extend the follow-up sessions, perhaps on a yearly basis, to reinforce the effects of the TSP on the participants. Finally, the TSP may benefit some students with particular characteristics more than the others; comparisons of the TSP’s effects on different populations of medical students would help the school leaders to prioritize the stress intervention program.

### The strengths and weaknesses of this study

The strengths of this study included the following factors. First, the participants in the pilot study provided useful feedbacks in making the TSP fit better with students’ curriculum. Second, the participation rate was high, and all participants completed the surveys. Third, the instruments used were validated for medical students. Lastly, the training effects were examined directly after the training and after a six-month follow-up, which demonstrated the immediate and lasting effects of the training. On the other hand, this study has limitations, including the lack of randomization at individual level and the prolonged time of collecting surveys at T2 due to the Covid-19 outbreak. In addition, we have not measured the status of psychological well-being of the students as indirect outcomes of the intervention, which may be of interest to all medical educators.

## Conclusion

Our findings have implications for stress interventions for medical students and identify areas for future investigations. Stress mindset and coping strategies could be modified by a practical intervention to be more positive and adaptive, although it is likely that continual reinforcement may be needed to maintain the TPS effects. This training could be replicated in other universities in Vietnam because of its clear procedures and validated instruments. The TSP adds to the existing literature of stress intervention programs recommended to medical students during their crucial stage of personal and professional development.

### Electronic supplementary material

Below is the link to the electronic supplementary material.

**Additional file 1:** Transforming Stress Program’s Components

## Data Availability

The datasets generated and/or analyzed during the current study are not publicly available due to the data protection policy of University of Medicine and Pharmacy, but are available from the corresponding author on reasonable request.
