# Supplementary material for: Transforming stress program on medical students’ stress mindset and coping strategies: a quasi-experimental study
Source: BMC Med Educ. 2023 Aug 18;23:587. doi: 10.1186/s12909-023-04559-9 (PMC10439558; doi:10.1186/s12909-023-04559-9)
Supplement: Supplementary file 1 — Additional file 1: Transforming Stress Program’s Components [file 12909_2023_4559_MOESM1_ESM.pdf]

Additional file

File name: Additional file 1: Transforming Stress Program's Components

File format: PDF

Description: The file provides the detailed information of the training course content of each session.

Additional file 1: Transforming Stress Program's Components

	Course content
1	Explaining a definition of Stress<br>Analyzing CBT model applied in Stress Management<br>Labeling triggers of Stress<br>Demonstrating and practicing changing mindset to appraisal stress
2	Categorizing stress coping strategies<br>Making plan to do the personal coping strategies<br>Demonstrating and practicing deep breathing<br>Demonstrating and practicing post stress self-reflection
3	Providing guidance in taking care of the body and the mind<br>Providing guidance in establishing a new habit<br>Making plan to do the self-care<br>Asking for help in time of need
4	*Course follow up session*<br>Add on: Demonstrating how to read stress personal report
5	*Course follow up session*<br>Add on: Demonstrating and practicing gratitude journal
